# Revealing the genotype-phenotype correlations of congenital hypothyroidism in Yunnan Province, Southwest China

**DOI:** 10.3389/fendo.2025.1640108

**Published:** 2025-11-17

**Authors:** Yinhong Zhang, Shiyu Wang, Aoyu Li, Wenjing Zhao, Fengyu Xia, Ying Chan, Junyue Lin, Xiaoyan Zhou, Suyun Li, Na Feng, Baosheng Zhu, Li Li

**Affiliations:** 1 Department of Medical Genetics, NHC Key Laboratory of Healthy Birth and Birth Defect Prevention in Western China, Yunnan Provincial Key Laboratory for Birth Defects and Genetic Diseases, The Afliated Hospital of Kunming University of Science and Technology, The First People’s Hospital of Yunnan Province, Kunming, China; 2 School of Medicine, Kunming University of Science and Technology, Kunming, China; 3 Department of Pediatrics, The Affiliated Hospital of Kunming University of Science and Technology, The First People’s Hospital of Yunnan Province, Kunming, China

**Keywords:** congenital hypothyroidism, thyroid hormone, genotype-phenotype, next-generation sequencing, thyroid morphology, clinical outcome

## Abstract

**Context:**

Congenital hypothyroidism (CH) is a congenital endocrine disorder with diverse clinical presentations. The genotype–phenotype relationship has recently become a focal point in genetic etiology research on CH.

**Objective:**

To explore the correlation between genetic variants and the clinical and biochemical characteristics of patients with CH in Yunnan Province, Southwest China.

**Methods:**

A retrospective analysis of 117 Yunnan-origin CH patients was conducted. Target regions capture next-generation sequencing (NGS) was used to screen for variations in all exons and their exon–intron boundaries in 27 CH-related genes. Patients were categorized into groups based on genetic variations; clinical outcomes were assessed through standardized follow-up.

**Results:**

Among the 117 CH patients, 91 carried gene variations related to CH, yielding a detection rate of 77.8%. Notably, variations in *DUOX2*, *DUOXA2*, and *TG* was most prevalent. Specifically, *DUOX2* gene variations were found in 67 CH patients; these mutations encompassed 47 variant types, with K530X, R885L, and R1110Q being the most common in the Chinese cohort. CH patients exhibiting goiter and thyroid dysgenesis required a higher initial levothyroxine (L-T4) dose. As the number of gene variants increased, thyroid morphology gradually shifted toward “goiter” and “dysgenesis”. No significant differences were observed in biochemical characteristics or clinical outcomes among genetic variant groups.

**Conclusions:**

This study provides valuable insights into the genetic landscape of CH in Yunnan Province, highlighting the importance of genes associated with thyroid dyshormonogenesis. Genotype cannot effectively be used to predict CH phenotype and prognosis. Standardized treatment and follow-up are crucial for positive outcomes in CH children.

## 1 Introduction

Congenital hypothyroidism (CH) is a congenital endocrine disorder possibly caused by abnormalities in thyroid differentiation, migration, or development or insufficient synthesis of thyroid hormones (1). The estimated incidence of sporadic CH (outside iodine-deficient areas) is approximately 1/2000–1/3000 (2, 3). CH typically presents as prolonged physiological jaundice, abdominal distention, constipation, umbilical hernia, feeding difficulties, low-pitched crying, and distinctive facial features (4). However, due to the influence of maternal thyroid hormones during fetal development, affected individuals often do not exhibit evident symptoms at birth, potentially leading to underdiagnosis (5). Thyroid hormones not only impact a child’s physical growth and development but also play a critical role in intellectual development (6). Without timely treatment, newborns with CH may have irreversible physical and intellectual developmental deficits (7).

Based on the site at which the pathology causes thyroid dysfunction, CH can be classified into primary CH and central hypothyroidism. Central hypothyroidism may occur with isolated TSH deficiency but is more commonly associated with congenital hypopituitarism (8). Traditional newborn screening can detect only primary CH associated with elevated thyroid-stimulating hormone (TSH) levels (9). CH can also be categorized as permanent CH (PCH) and transient CH (TCH) according to its prognosis (10). PCH is mostly caused by thyroid absence or underdevelopment, leading to a continuous deficiency of thyroid hormones in affected individuals, who require lifelong levothyroxine (L-T4) replacement therapy. The etiology of TCH is often linked to maternal iodine deficiency, neonatal iodine exposure, maternal autoimmune thyroid disease, premature infant thyroid immaturity, etc., leading to temporary insufficient thyroid hormone secretion, which typically recovers by the age of 2–3 years, with discontinuation of L-T4 therapy (11, 12).

Despite considerable progress in the screening, diagnosis, and treatment of CH (13), the underlying mechanisms remain unclear. CH pathogenesis can be attributed to thyroid dysgenesis (TD) and dyshormonogenesis (DH). Recent studies have identified genetic defects associated with CH, with more than 40 associated genes, including *TSHR*, *TITF2*, *NKX2 (TITF1)*, *PAX8*, *NKX2-5*, *DUOX2*, *GLIS3*, *TUBB1*, *CDCA8*, *JAG1*, *HHEX*, *HES1*, *HOXA3*, *EYA1*, *TPO*, *SLC5A5*, *DUOX1*, *DUOXA2*, *SLC26A4*, *TG*, *TRHR*, *TSHB*, *IGSF1*, *HESX1*, *TBL1X*, *IYD*, *LEPR*, *PROP1*, *POU1F1*, *LHX3*, and*LHX4*, among others (14).

Furthermore, research indicates strong population-specific differences in variants and phenotypes of CH. However, the relationships between genotype and thyroid development, clinical outcome, and L-T4 dose remain unclear. Therefore, further studies are needed such that the underlying genetic causes of CH can be fully understood. In this study, we comprehensively and systematically screened mutated genes through next-generation sequencing (NGS), aiming to investigate the correlation between the genetic mutation status of CH-related genes and phenotypes in Yunnan Province, Southwest China.

## 2 Methods

### 2.1 Patients

A total of 117 Yunnan-origin CH patients treated at the First People’s Hospital of Yunnan Province from January 2016 to January 2019 were retrospectively analyzed. These CH patients were diagnosed at an age ranging from 15 to 30 days; 54 males and 63 females were included. The inclusion criteria were as follows: (i) infants with negative thyroid autoantibody test results; (ii) lacking other congenital diseases or chromosomal abnormalities; and (iii) sporadic and non-consanguineous cases. The exclusion criteria were as follows: (i) central hypothyroidism or related syndromes and (ii) autoimmune diseases or family history thereof. The study area (Yunnan Province) has been classified as an iodine-sufficient region following implementation of the national universal salt iodization program.

This study was reviewed and approved by the Medical Ethics Committee of the First People’s Hospital of Yunnan Province (No. KHLL2023-KY037). Signed informed consent was obtained from the parents of all patients. The research protocol complied with the seventh revision of the Helsinki Declaration (2013).

### 2.2 Newborn screening and laboratory diagnostic criteria for CH

The newborns were all screened at the Yunnan Province Newborn Screening Center. Within 72 hours to 20 days after birth, dried blood spot samples were collected from the heel of the newborns. These samples were then transported to the screening center laboratory through a cold chain system, and testing was completed within 3 days. TSH values were measured using a time-resolved fluorescence immunoassay (Perkin Elmer), with a cutoff value set at ≥8 μmol/L. Newborns with TSH screening results exceeding this positive cutoff value were recalled for serum thyroid function tests. Thyroid function was assessed in the patient’s serum using five different parameters, and values were compared to established normal reference ranges (TSH: 0.87∼6.15 mIU/L, T4: 77.8∼170.0 nmol/L, FT4: 12.0∼18.6 pmol/L, T3: 1.80∼3.68 nmol/L, FT3: 5.1∼8.0 pmol/L). A diagnosis of CH was made when the patient’s serum FT4 level fell below the minimum threshold and the TSH level exceeded the maximum detectable threshold.

### 2.3 Ultrasound measurement of the thyroid in CH patients

The long diameter (L), wide diameter (W), and thick diameter (T) of the thyroid were measured using a SIEMENS-ACUSON-S2000 color Doppler ultrasound instrument. The probe frequency was set at 9~18 MHz, and the color scale parameter was conventionally set at 7 cm/s. The ultrasound examination procedure was as follows. After the child was calm or asleep, the child was placed in the supine position with appropriate neck elevation to fully expose the neck. The ultrasound probe was positioned in front of the neck, and during the examination, it was placed just below the thyroid cartilage. Transverse and longitudinal scans using two-dimensional ultrasound were initially conducted to carefully observe the thyroid’s size, shape, capsule, and internal echogenicity. The largest diameter of the right and left thyroid lobes and the isthmus was measured, and the formula V = 0.479LWT/1000 was used to calculate the volume of both lobes (15). Color Doppler flow imaging (CDFI) was used to assess the degree and distribution of blood flow within the thyroid. By comparing the thyroid volume of the CH patients with that of healthy infants of corresponding ages, the thyroids of the CH patients were categorized into normal, dysgenesis (agenesis or athyreosis, ectopy, orthotopic hypoplasia and hemiagenesis) and goiter groups.

### 2.4 Target regions capture, sequencing and pathogenicity analysis of gene variations

Five milliliters of peripheral blood was collected from the patients and their parents. Genomic DNA (gDNA) was extracted according to the kit manufacturer’s standard procedure (MagPure Buffy Coat DNA Midi KF Kit, MAGEN BIOTECH). The gDNA was broken into 100–500 bp fragments by Shearing Enzyme Premix Reagent (ENZYMATICS), and 200–300 bp fragments were subsequently collected by magnetic beads (Vazyme DNA Clean Beads, VAZYME). An “A” base was added at the 3’ overhang after the end of the repair process to ensure that the fragments could pair with the “T” base with a special adapter, after which a single individual DNA library was constructed after Linker-mediated-PCR and purification. The library was enriched for 16 h at 65 °C by array hybridization (KAPA Hyper Exome, ROCHE), followed by elution and post-capture amplification. The products were subsequently subjected to analysis with a CaliperGX_1000 Kit (Caliper Life Sciences) and BMG to estimate the magnitude of enrichment. The qualified products were pooled and quantified according to different library sizes. Then, single strands of the library products were prepared for circularization, and DNB was generated. Finally, the products were sequenced with PE100 + 10 using an MGISEQ-2000.

To detect potential variants in the patients, bioinformatics processing and data analysis were performed after receiving the primary sequencing data. Comprehensive analysis was conducted to screen for mutations in 27 genes associated with CH, including *HESX1*, *LHX3*, *LHX4*, *SOX3*, *OTX2*, *PROP1*, *POU1F1*, *TRHR*, *TSHB*, *LEPR*, *TPO*, *TSHR*, *TBL1X*, *IYD*, *PAX8*, *NKX2-1*, *FOXE1*, *IGSF1*, *NKX2-5*, *JAG1*, *GLIS3*, *CDCA8*, *TG*, *DUOX2*, *DUOXA2*, *SLC5A5*, and *SLC26A4*. Variants located in regions with <10× effective coverage were considered low confidence. Such sites were either re-evaluated by repeat sequencing or excluded from downstream analyses unless validated independently by Sanger sequencing. For cases with missing data at specific loci, these positions were treated as unavailable and not used for genotype–phenotype correlation analyses. Only high-confidence variants confirmed by both NGS and, when necessary, Sanger sequencing were included in the final dataset. The “clean reads” (with a length of 90 bp) were derived from targeted sequencing and filtering and subsequently aligned to the human genome reference (hg19) using the Burrows Wheeler Aligner (BWA) MultiVision software package. All SNVs and indels were filtered and estimated via multiple databases, including the 1000 Genomes Project (1KGP), Genome Aggregation Database (gnomAD), Human Gene Mutation Database (HGMD) and Clinvar. We used dbNSFP, which contains seven well-established in silico prediction programs (scale-invariant feature transform (SIFT), Polyphen2, LRT, Mutation Taster, and PhyloP), to predict the effect of missense variants. The pathogenicity of mutations was interpreted based on American College of Medical Genetics and Genomics (ACMG) guidelines (16). All mutations and potential pathogenic variants were validated using conventional Sanger sequencing methods.

### 2.5 Follow-up and management

Treatment, regular follow-up, and monitoring of confirmed cases were conducted by pediatric endocrinologists from the Newborn Screening Center. Several highly compliant patients received standardized outpatient follow-up and treatment, including assessments of height, weight, head circumference, Gesell development schedules, and L-T4 doses at 1, 2, and 3 years of age. All CH children temporarily stopped L-T4 treatment at the age of 2 years as part of the standardized treatment protocol. Thyroid function was tested after one month. If TSH and FT4 levels were abnormal, the original treatment dosage was resumed. These CH children were diagnosed with PCH and continued to receive regular treatment and follow-up. CH children who stopped L-T4 treatment and maintained normal thyroid function continuously for more than one year were diagnosed with TCH.

### 2.6 Statistical analyses

Statistical analysis was conducted using SPSS 21.0 software and GraphPad Prism 8 software. We summarized normally distributed quantitative variables using means and standard deviations (means ± SDs). For nonnormally distributed variables, we employed medians with interquartile intervals (Q1-Q3). Count data are expressed as the number of patients (percentages) [N (%)]. The normality of continuous data was assessed using the Shapiro–Wilk test. For normally distributed data with homogeneity of variance, the independent samples t-test or one-way analysis of variance (ANOVA) followed by Bonferroni *post hoc* correction was used for intergroup comparisons. The Kruskal–Walli’s test was used for nonnormally distributed continuous data. Comparison of count data was performed using the chi-square test or Fisher’s exact test. A *P-*value less than 0.05 was considered to indicate statistical significance. Graphs were generated using GraphPad Prism 8 software.

## 3 Results

### 3.1 Clinical and biochemical characteristics and phenotypes of CH

Our cohort comprised a total of 117 patients with CH, 91 of whom were identified as carriers of variations in CH-related genes; these patients included 46 males and 45 females. No statistically significant differences were detected between the male and female patients in terms of ethnic distribution, birth age, birth weight, birth height, age at diagnosis, maternal thyroid function, TSH levels at newborn screening (NBS), diagnostic serum TSH levels, diagnostic serum FT4 levels, initial L-T4 doses, thyroid morphology, or clinical outcome (**Table 1**).

**Table 1:** Clinical and biochemical characteristics and clinical outcome of 91 children with CH.

Characteristics	Male (N = 46) N (%)	Female (N = 45) N (%)	Total (N = 91) N (%)	*P* value
Ethnic group				
Han	39 (50.0)	39 (50.0)	78 (85.7)	0.797
Minority	7 (53.8)	6 (46.2)	13 (14.3)	
Birth age (w)				
<37	3 (100.0)	0 (0)	3 (3.3)	0.242
37∼42	43 (48.9)	45 (51.1)	88 (96.7)	
≥42	0 (0)	0 (0)	0 (0)	
Birth weight (g)				
<2500	4 (66.7)	2 (33.3)	6 (6.6)	0.693
2500∼4000	42 (49.4)	43 (50.6)	85 (93.4)	
>4000	0 (0)	0 (0)	0 (0)	
Birth height (cm)				
<50	15 (55.6)	12 (44.4)	27 (29.7)	0.535
≥50	31 (48.4)	33 (51.6)	64 (70.3)	
Age at diagnosis(days)				
≤14	5 (71.4)	2 (28.6)	7 (7.7)	0.348
15∼21	18 (50.0)	18 (50.0)	36 (39.6)	
22∼30	10 (38.5)	16 (61.5)	26 (28.6)	
>30	13 (59.1)	9 (40.9)	22 (24.2)	
Maternal thyroid function				
Normal	38 (49.4)	39 (50.6)	77 (84.6)	0.67
Clinical hypothyroidism	6 (66.7)	3 (33.3)	9 (9.9)	
Subclinical hypothyroidism	2 (40.0)	3 (60.0)	5 (5.5)	
TSH levels at NBS (Reference value: <8 mIU/L) median (Q1–Q3)	29.50 (18.42, 100.00)	28.00 (14.55, 101.50)	28.20 (15.50, 101.50)	0.683
Diagnostic serum TSH levels (Reference value: 0.87–6.15 mIU/L) median (Q1–Q3)	50.10 (15.82, 100.00)	71.90 (18.28, 100.00)	55.10 (17.00, 100.00)	0.679
Diagnostic serum FT4 levels (Reference value: 12.0–18.6 pmol/L) mean ± SD	6.99 ± 3.15	6.00 ± 2.46	6.50 ± 2.86	0.1
Initial L-T4 doses (μg/day) median (Q1–Q3)	25.0 (12.5, 37.5)	25.0 (12.5, 37.5)	25.0 (12.5, 37.5)	0.51
Thyroid morphology				
Normal	22 (56.4)	17 (43.6)	39 (42.8)	0.387
Goiter	22 (48.9)	23 (51.1)	45 (49.4)	
Dysgenesis	2 (28.6)	5 (71.4)	7 (7.7)	
Clinical outcome				
PCH	37 (47.4)	41 (52.6)	78 (85.7)	0.248
TCH	9 (69.2)	4 (30.8)	13 (14.3)	

CH, congenital hypothyroidism; N, number; TSH, thyroid-stimulating hormone; NBS, newborn screening; FT4, free thyroxine; L-T4, levothyroxine; PCH, permanent CH; TCH, transient CH.

Among the 91 patients, 39 had a normal thyroid, 45 had an enlarged thyroid, and 7 exhibited thyroid dysgenesis; 4 had thyroid agenesis, and 3 had orthotopic hypoplasia. After more than 2 years of treatment and follow-up, 78 cases were classified as PCH, whereas 13 cases were classified as TCH.

### 3.2 Genetic variation distribution in 91 CH patients

**Figure 1:**
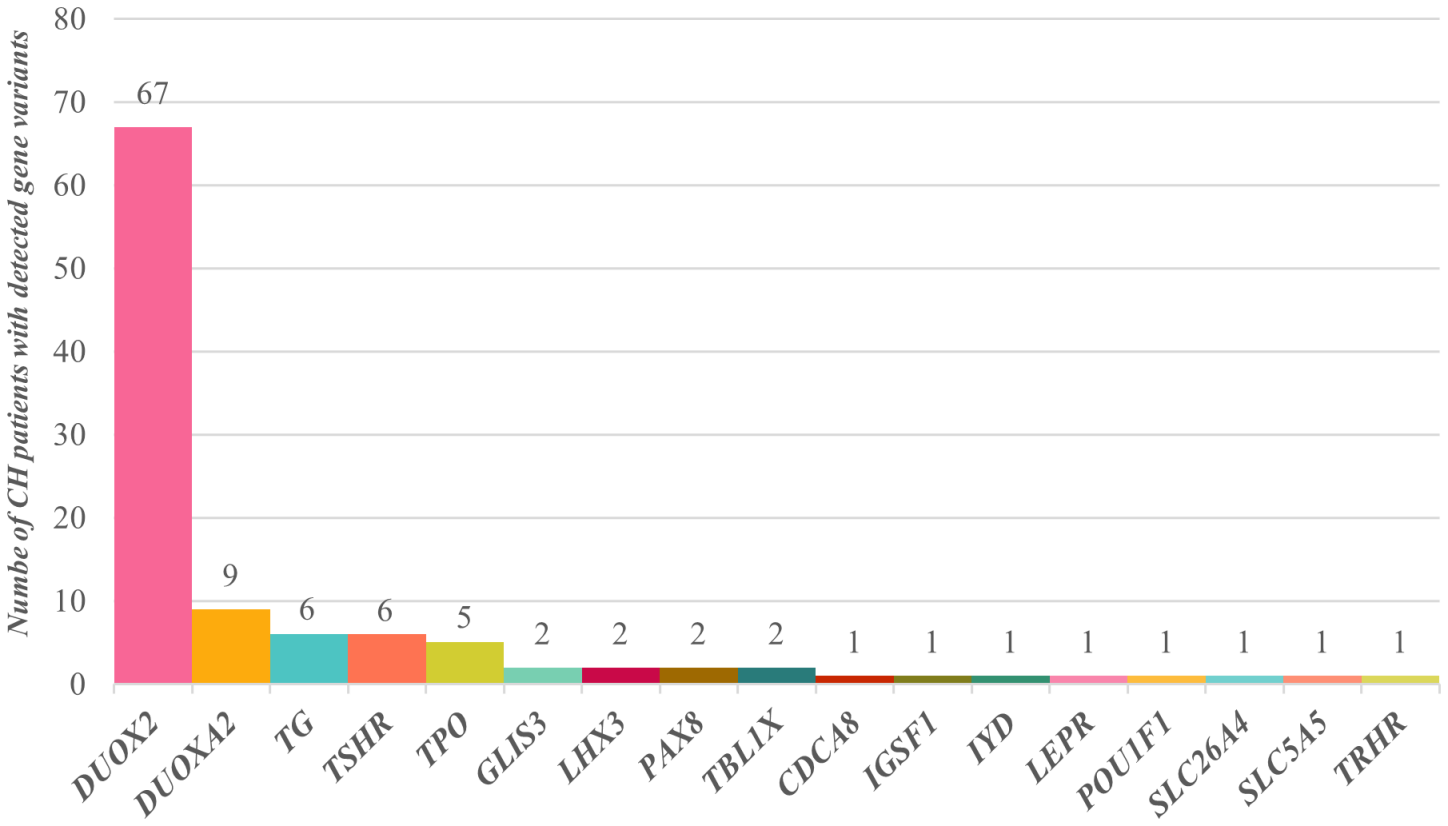
Distribution of CH-related gene variants in 91 children. Sixty-seven children had *DUOX2* gene variants, 9 had *DUOXA2* gene variants, 6 had *TG* gene variants, 6 had *TSHR* gene variants, and 5 had *TPO* gene variants. Variants in the *GLIS3*, *LHX3*, *PAX8*, and *TBL1X* genes were each detected in 2 children, and *CDCA8*, *IGSF1*, *IYD*, *LEPR*, *POU1F1*, *SLC26A4*, *SLC5A5*, and *TRHR* gene variants were each detected in a single child.

The detailed general information, biochemical test results, treatment results, clinical outcomes, and family molecular test results for the 91 patients are provided in **Supplementary Table 1**. Among them, 67 patients carried *DUOX2* gene variations, 9 carried *DUOXA2* gene variations, 6 carried *TG* gene variations, 6 carried *TSHR* gene variations, 5 carried *TPO* gene variations, and 2 each carried variations in the *GLIS*3, *LHX3*, *PAX8*, and *TBL1X* genes. Additionally, 1 patient each carried variations in the *CDCA8*, *IGSF1*, *IYD*, *LEPR*, *POU1F1*, *SLC26A4*, *SLC5A5*, and *TRHR* genes (**Figure 1**). Clearly, *DUOX2* gene variations had the highest frequency in this cohort, accounting for 61.5% (67/109) of the total. The next most common variations involved *DUOXA2*, *TG*, *TSHR*, and *TPO*, which were associated with 8.3% (9/109), 5.5% (6/109), 5.5% (6/109), and 4.6% (5/109), respectively. A total of 47 variant types and 117 variant frequencies were identified in the *DUOX2* gene, with c.1588A>T (p.K530X), c.2654G>T (p.R885L), c.3329G>A (p.R1110Q), c.2048G>T (p.R683L), c.3693 + 1G>T, c.2921G>A (p.R974H), and c.4027C>T (p.L1343F) accounting for 12.8%, 10.2%, 10.2%, 6.8%, 5.9%, 5.1%, and 4.2%, respectively. The most common *DUOXA2* gene variant was c.738C>G (p.Y246X), accounting for 70.0% (7/10) of the total cases. Notably, digenic variants were found in 11 patients, and oligogenic variants were found in 3 patients. Among gene combinations, *DUOX2*+*TG* was the most common, accounting for 27.3% (3/11).

### 3.3 Analyses of relationships between thyroid morphology and biochemical/clinical characteristics

**Figure 2:**
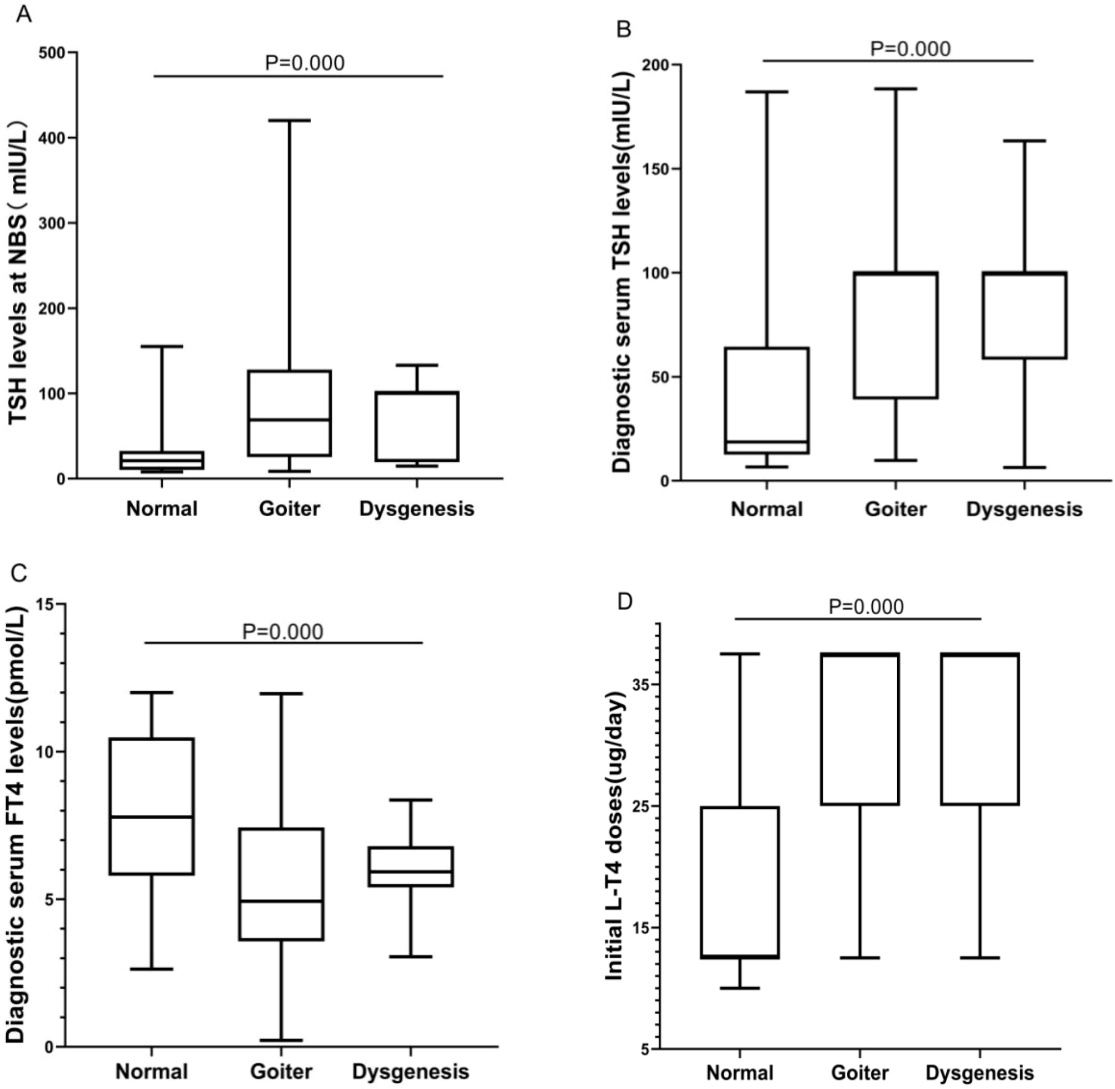
Differences in biochemical results and initial L-T4 doses among “normal”, “goiter”, and “dysgenesis” thyroid morphology groups. **(A)** The BOX plot chart shows the differences among the three groups regarding TSH levels at NBS. **(B)** The BOX plot chart shows the differences among the three groups regarding diagnostic serum TSH levels. **(C)** The BOX plot chart shows the differences among the three groups regarding diagnostic serum FT4 levels. **(D)** The BOX plot chart shows the differences among the three groups regarding the initial L-T4 doses. A *P* value <0.001 was considered to indicate an extremely significant difference.

The CH patients were grouped according to thyroid morphology, and we conducted statistical analysis of differences in biochemical test results and clinical outcomes among the groups. We found statistically significant differences (*P*< 0.001) among the “normal”, “goiter”, and “dysgenesis” groups in terms of TSH levels at NBS, diagnostic serum TSH levels, and diagnostic serum FT4 levels. The “goiter” group and the “dysgenesis” group exhibited higher TSH levels at NBS and diagnostic serum TSH levels, corresponding to lower diagnostic serum FT4 levels. Moreover, the “goiter” group and the “dysgenesis” group required higher initial L-T4 doses than did the “normal” group. However, there was no statistically significant difference in clinical outcomes among the groups (*P*>0.05) (**Table 2**, **Figure 2**).

**Table 2:** Differences in biochemical and clinical characteristics of 91 children with CH among “normal”, “goiter”, and “dysgenesis” groups.

Characteristics	Normal (N = 39)	Goiter (N = 45)	Dysgenesis (N = 7)	*P* value
TSH levels at NBS (Reference value: <8 mIU/L) median (Q1–Q3); mean ± SD	21.00 (10.30, 32.40)	69.00 (25.25, 128.00)	80.41 ± 45.16	<0.001
Diagnostic serum TSH levels (Reference value: 0.87–6.15 mIU/L) median (Q1–Q3); mean ± SD	18.65 (12.56, 64.51)	100.00 (39.11, 100.00)	89.42 ± 47.87	<0.001
Diagnostic serum FT4 levels (Reference value: 12.0–18.6 pmol/L) mean ± SD	7.79 ± 2.61	5.46 ± 2.80	5.99 ± 1.61	<0.001
Initial L-T4 doses (μg/day) median (Q1–Q3)	12.5 (12.5, 25.0)	37.5 (25.0, 37.5)	37.5 (25.0, 37.5)	<0.001
Clinical outcome				
PCH	31	40	7	0.217
TCH	8	5	0	

CH, congenital hypothyroidism; N, number; TSH, thyroid-stimulating hormone; NBS, newborn screening; FT4, free thyroxine; L-T4, levothyroxine; PCH, permanent CH; TCH, transient CH.

### 3.4 Analyses of relationships between *DUOX2* genotypes and biochemical/clinical characteristics

**Figure 3:**
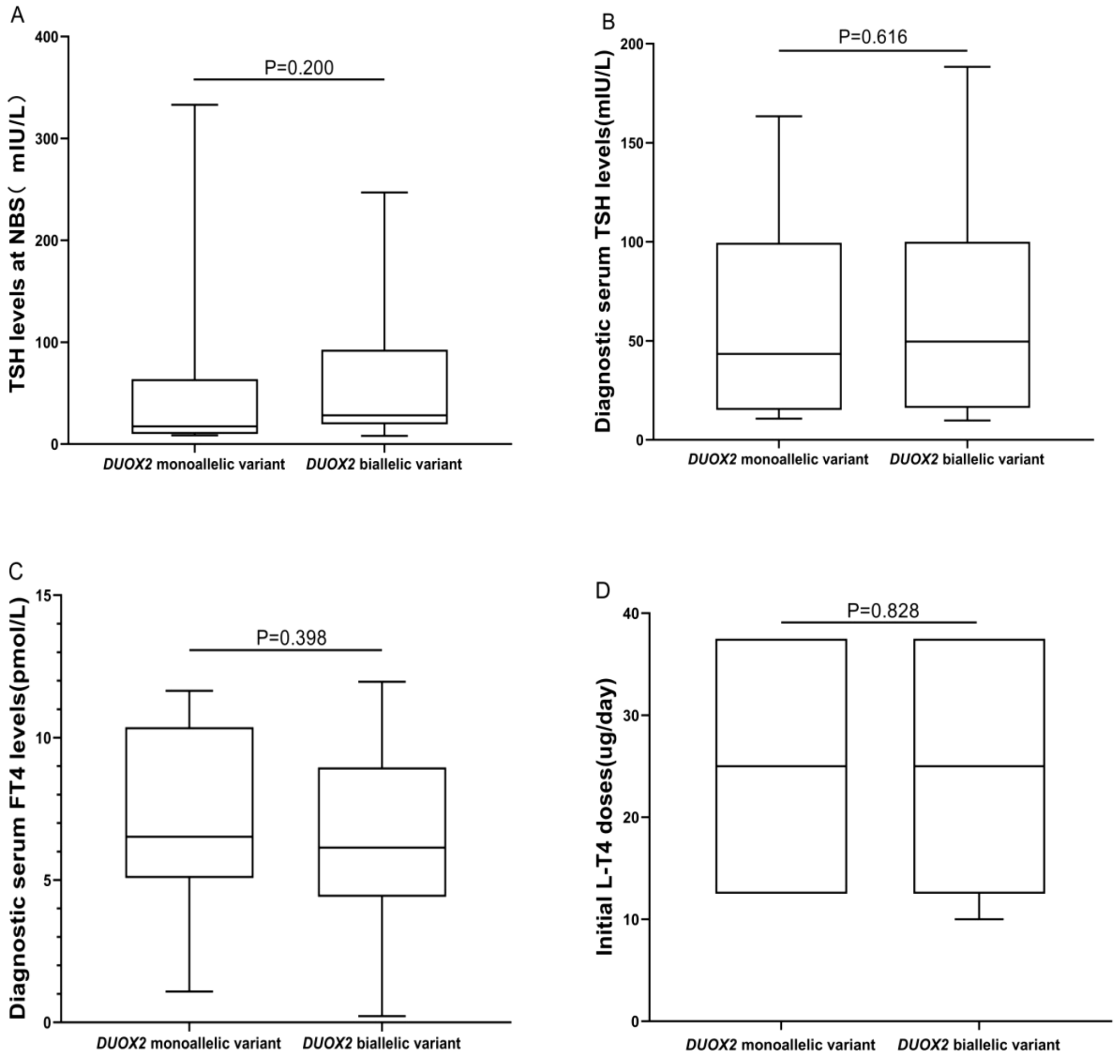
Differences in biochemical results and initial L-T4 doses between “*DUOX2* monoallelic variant” and “*DUOX2* biallelic variant” groups. **(A)** The BOX plot chart shows the differences between the *DUOX2* monoallelic and biallelic variant groups regarding TSH levels at NBS. **(B)** The BOX plot chart shows the differences between the *DUOX2* monoallelic and biallelic variant groups regarding diagnostic serum TSH levels. **(C)** The BOX plot chart shows the differences between the *DUOX2* monoallelic and biallelic variant groups regarding diagnostic serum FT4 levels. **(D)** The BOX plot chart shows the differences between the *DUOX2* monoallelic and biallelic variant groups regarding the initial L-T4 doses. *P*>0.05 was considered to indicate no statistical significance.

*DUOX2* genotypes were divided into monoallelic variant and biallelic variant groups, and statistical analysis was conducted to assess differences between these two groups in terms of biochemical results, initial L-T4 doses, thyroid morphology, and clinical outcomes. There were no significant differences in TSH levels at NBS, diagnostic serum TSH levels, diagnostic serum FT4 levels, initial L-T4 doses, or clinical outcomes (all *P*> 0.05). However, there was a significant difference in thyroid morphology between the two groups (*P* = 0.05). A greater proportion of patients in the “*DUOX2* monoallelic variant” group had normal thyroid morphology (50.00%, 10/20), whereas a greater proportion of patients in the “*DUOX2* biallelic variant” group had goiter (59.57%, 28/47) (**Table 3**, **Figure 3**).

**Table 3:** Differences in biochemical and clinical characteristics of 67 children with CH between “*DUOX2* monoallelic variant” and “*DUOX2* biallelic variant” groups.

Characteristics	*DUOX2* monoallelic variant (N = 20)	*DUOX2* biallelic variant (N = 47)	*P* value
TSH levels at NBS (Reference value: <8 mIU/L) median (Q1–Q3)	20.00 (9.83, 90.68)	28.20 (19.40, 92.80)	0.200
Diagnostic serum TSH levels (Reference value: 0.87–6.15 mIU/L) median (Q1–Q3)	40.65 (15.13, 99.50)	49.69 (16.12, 100.00)	0.616
Diagnostic serum FT4 levels (Reference value: 12.0–18.6 pmol/L) mean ± SD	7.06 ± 3.22	6.38 ± 2.85	0.398
Initial L-T4 doses (μg/day) median (Q1–Q3)	25.0 (12.5, 37.5)	25.0 (12.5, 37.5)	0.828
Thyroid morphology			
Normal	10	18	0.051
Goiter	7	28	
Dysgenesis	3	1	
Clinical outcome			
PCH	16	39	1.000
TCH	4	8	

CH, congenital hypothyroidism; N, number; TSH, thyroid-stimulating hormone; NBS, newborn screening; FT4, free thyroxine; L-T4, levothyroxine; PCH, permanent CH; TCH, transient CH.

### 3.5 Analyses of relationships between genotypes and clinical phenotypes

**Figure 4:**
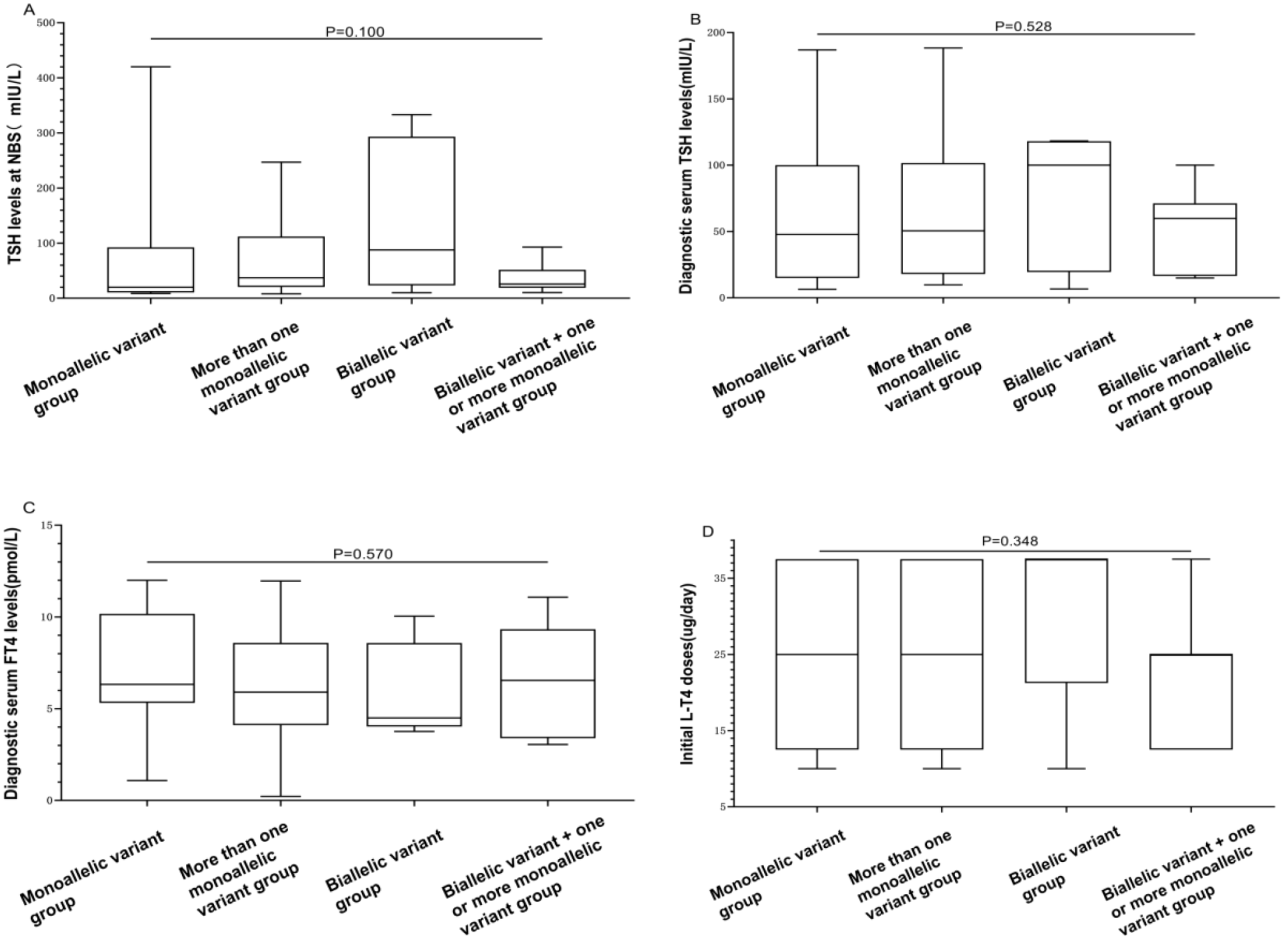
Differences in biochemical results and initial L-T4 doses among “monoallelic variant”, “more than one monoallelic variant”, “biallelic variant”, and “biallelic variant + one or more monoallelic variant” groups. **(A)** The BOX plot chart illustrates differences among the four groups concerning TSH levels at NBS. **(B)** The BOX plot chart displays differences among the four groups concerning diagnostic serum TSH levels. **(C)** The BOX plot chart shows differences among the four groups regarding diagnostic serum FT4 levels. **(D)** The BOX plot chart demonstrates differences among the four groups regarding the initial L-T4 doses. A *P* value > 0.05 was considered to indicate no statistically significant difference.

To analyze relationships between genotypes and clinical phenotypes, the 91 CH patients were divided into four groups based on the type of gene variant: those carrying only one monoallelic variant were classified as the “monoallelic variant” group; those with two or more monoallelic variants were in the “more than one monoallelic variant” group; those with only one biallelic variant were in the “biallelic variant” group; and those with one or more monoallelic variants in addition to one biallelic variant were in the “biallelic variant + one or more monoallelic variant” group. The numbers of patients in these four groups were 32, 6, 45, and 8, respectively. There were no significant differences among the four groups in terms of TSH levels at NBS, diagnostic serum TSH levels, diagnostic serum FT4 levels, initial L-T4 doses, or clinical outcomes (*P* = 0.100, 0.528, 0.570, 0.348, 0.684, respectively). However, there was a significant difference in thyroid morphology among the four groups (*P* = 0.003). The “monoallelic variant” group had a greater proportion of individuals with “normal” thyroid morphology, accounting for 53.1% (17/32) of the population. With an increase in the number of gene variants, the thyroid morphology gradually shifted toward “goiter” and “dysgenesis” (**Table 4**, **Figure 4**).

**Table 4:** Differences in biochemical and clinical characteristics of 91 children with CH based on gene variant combinations.

Characteristics	Monoallelic variant (N = 32)	More than one monoallelic variant (N = 6)	Biallelic variant (N = 45)	Biallelic variant + one or more monoallelic variant (N = 8)	*P* value
TSH levels at NBS (Reference value: <8 mIU/L) median (Q1–Q3); mean ± SD	20.00 (10.53, 92.38)	137.71 ± 135.96	37.20 (20.50, 112.00)	25.90 (18.98, 51.75)	0.100
Diagnostic serum TSH levels (Reference value: 0.87–6.15 mIU/L) median (Q1–Q3); mean ± SD	47.79 (14.93, 100.00)	100.00 (19.42, 118.09)	50.50 (17.95, 101.61)	51.18 ± 31.67	0.53
Diagnostic serum FT4 levels (Reference value: 12.0–18.6 pmol/L) mean ± SD	7.05 ± 2.98	5.84 ± 2.60	6.19 ± 2.81	6.58 ± 2.99	0.57
Initial L-T4 doses (μg/day) median (Q1–Q3); mean ± SD	25.0 (12.5, 37.5)	37.5 (21.2, 37.5)	25.0 (12.5, 37.5)	21.88 ± 8.84	0.35
Thyroid morphology					
Normal	17	1	17	4	0.003
Goiter	9	5	28	3	
Dysgenesis	6	0	0	1	
Clinical outcome					
PCH	27	6	39	6	0.684
TCH	5	0	6	2	

CH, congenital hypothyroidism; N, number; TSH, thyroid-stimulating hormone; NBS, newborn screening; FT4, free thyroxine; L-T4, levothyroxine; PCH, permanent CH; TCH, transient CH.

### 3.6 Growth and development of 25 children with CH of different genotypes

Among the 91 CH patients, 25 underwent standardized physical and intelligence assessments at the ages of 1, 2, and 3 years. The clinical outcomes included 23 cases of PCH and 2 cases of TCH. The physical development (height, weight, head circumference) and intellectual development (gross motor skills, fine motor skills, adaptive behavior, language, and personal-social behavior) of these patients were within the normal range, showing no significant differences compared to those of same-age children. The patients were divided into three groups based on the types of gene variants they carried. At the same monitoring time points, there were no significant differences in the above indicators among the patients with different gene variant types (all *P*> 0.05). Moreover, no significant differences in L-T4 doses at the maintenance level at the age of 3 years were detected among the three groups (all *P*> 0.05). A subset of 25 patients had complete standardized follow-up records at 1, 2, and 3 years; others were excluded due to incomplete data or drop-out (**Table 5**).

**Table 5:** Growth and development of 25 CH children with different gene variant combinations at 1 to 3 years of age.

Groups	N	Height(cm)	Weight(kg)	Head circumference(cm)	Gross motor skills	Fine motor skills	Adaptive behavior	Language	Personal-social behavior	Maintenance L-T4 doses(μg/day)
Monoallelic variant										
1 Y*^a^*	14	75.3 ± 1.2	9.9 ± 0.7	46.2 ± 1.0	89.3 ± 5.3	97.1 ± 6.0	93.6 ± 4.3	89.0 ± 6.6	93.4 ± 4.6	—
2Y*^b^*	14	87.3 ± 1.3	12.0 ± 0.8	48.1 ± 0.8	95.3 ± 5.0	93.7 ± 5.0	94.1 ± 6.4	89.1 ± 6.4	98.1 ± 4.3	—
3Y*^c^*	14	96.5 ± 1.2	14.1 ± 0.8	48.7 ± 0.9	93.0 ± 7.0	96.6 ± 5.6	92.9 ± 6.2	91.6 ± 4.8	95.3 ± 4.9	38.6 ± 17.6*^d^*
Biallelic variant										
1Y*^a^*	8	75.3 ± 1.0	9.4 ± 0.6	46.1 ± 1.4	94.6 ± 5.3	92.5 ± 6.7	90.0 ± 7.1	86.4 ± 7.6	91.3 ± 3.9	—
2Y*^b^*	8	88.4 ± 1.5	12.6 ± 1.4	47.7 ± 1.0	90.3 ± 4.6	92.1 ± 7.5	98.5 ± 6.6	87.3 ± 10.2	94.8 ± 7.8	—
3Y*^c^*	8	96.9 ± 1.6	14.1 ± 0.9	48.2 ± 1.0	98.5 ± 6.2	97.6 ± 6.0	94.8 ± 5.5	88.0 ± 11.3	94.3 ± 8.3	38.9 ± 13.1*^d^*
Oligogenic variant										
1Y*^a^*	3	75.7 ± 0.3	9.6 ± 0.6	47.5 ± 0.4	88.0 ± 4.0	95.0 ± 6.2	94.7 ± 4.9	89.7 ± 8.1	95.7 ± 4.9	—
2Y*^b^*	3	87.5 ± 1.8	12.6 ± 0.6	49.0 ± 0.2	93.0 ± 3.6	92.7 ± 9.0	94.3 ± 5.0	95.3 ± 4.6	97.3 ± 7.2	—
3Y*^c^*	3	96.0 ± 1.1	14.7 ± 1.2	49.8 ± 0.4	99.7 ± 9.3	90.3 ± 8.0	91.0 ± 9.2	92.0 ± 6.6	93.3 ± 8.5	33.3 ± 14.4*^d^*

CH, congenital hypothyroidism; N, number; Y, years.

*^a^*At the age of 1 year, there were no statistically significant differences among the various observed indicators in the CH patients, with respective *P*-values of 0.841, 0.241, 0.153, 0.06, 0.274, 0.265, 0.661, and 0.313.

*^b^*At the age of 2 years, there were no statistically significant differences among different observed indicators in the CH patients, with respective *P*-values of 0.244, 0.427, 0.077, 0.08, 0.846, 0.303, 0.316, and 0.443.

*^c^*At the age of 3 years, there were no statistically significant differences among different observed indicators in the CH patients, with respective *P*-values of 0.553, 0.552, 0.055, 0.141, 0.207, 0.652, 0.532, and 0.869.

*^d^*At the age of 3 years, there was no statistically significant difference in the maintenance L-T4 doses of the CH patients, with a *P*-value of 0.625.

## 4 Discussion

In this study, we employed targeted region capture NGS to analyze mutations in 27 CH-related genes in a comprehensive manner, aiming to unravel the molecular and clinical characteristics of 91 Chinese children with CH from Yunnan Province.

With widespread application of NGS, molecular research reports on CH have been increasing both in China and abroad. Application of genome sequencing technology for newborn screening is gradually becoming a future development trend (17–19). However, despite significant discoveries regarding CH-related genes, linking these genotypes to L-T4 treatment dosage and clinical outcomes poses a complex challenge. Our study detected relevant gene variations in 91 of 117 CH children from Yunnan, yielding a detection rate of 77.7% (91/117). No statistically significant differences were observed in the demographic or clinical characteristics between male and female patients, underscoring the uniform distribution of CH-related characteristics in this cohort. The five most commonly mutated genes were *DUOX2*, *DUOXA2*, *TG*, *TSHR*, and *TPO.* Notably, a subset of CH patients eventually exhibited TCH, constituting 14.3% (13/91) of the clinical outcomes. This finding is consistent with previous reports, as the clinical outcomes of CH detected through newborn screening continue to be primarily represented by PCH (11, 20).

CH is predominantly associated with TD and DH. DH accounts for approximately 35% of CHs, and a genetic cause is identified in 50% of patients; TD accounts for approximately 65% of CHs, and a genetic cause is identified in less than 5% of patients (21). Genes related to TD include *PAX8*, *TSHR*, *NKX2-1*, *FOXE1*, *NKX2-5*, *TUBB1*, and *HHEX*; these genes are often inherited in an autosomal dominant (AD) or autosomal recessive (AR) manner (22–25). On the other hand, genes associated with DH include *DUOX2*, *DUOXA2*, *TPO*, *TG*, *SLC26A4*, *SLC5A5*, and *IYD*, which are typically inherited in an AR-related manner (14, 26). In previous reports, the majority of CH cases were attributed to TD, as characterized by features such as agenesis or athyreosis, ectopy, orthotopic hypoplasia and hemiagenesis (27, 28). However, in our cohort, patients with a normal or enlarged thyroid constituted the overwhelming majority, accounting for 92.2% (84/91). Furthermore, among the five most common genetic variations in our cohort, *DUOX2*, *DUOXA2*, *TG*, and *TPO* mutations were associated with DH. Given the diverse ethnicities, geographical environments, dietary patterns and genetic backgrounds in China, existing research reports show slight variations in the ranking of gene variations (29, 30). Nevertheless, overall, genes related to DH dominate (31). In contrast, types of genetic variants associated with CH have been associated mainly with TD in Western populations (32, 33). It is undeniable that the mechanisms underlying CH occurrence display significant racial and ethnic variations.

In our cohort, 67 CH patients (61.5%) were identified to harbor *DUOX2* variants, revealing a higher *DUOX2* gene mutation rate, consistent with the findings of scholars in other regions of China, Korea, and Japan (34–37). This finding suggests that *DUOX2* gene variants might be a primary genetic factor involved in the occurrence of CH in East Asian populations. Some studies from other countries report that variants in the *TPO* or *TG* gene are the most common cause of synthesis disorders, possibly linked to racial and regional variations (38–40). The variants c.1588A > T (p.K530X), c.3329G > A (p.R1110Q), and c.2654G>T (p.R885L) were most common in our cohort, with K530X previously reported to have a higher variant frequency in the Chinese population, emphasizing this significant characteristic. As a member of the NADPH oxidase family, *DUOX2* plays a pivotal role in the generation of H_2_O_2_ in the thyroid, participating in a critical step in thyroid hormone synthesis. *DUOX2* variations commonly impede iodination of thyroglobulin, consequently hampering synthesis of thyroid hormones (41). However, recent research has revealed that certain *DUOX2* variants may also be associated with TD (15). In our study, three patients with either monoallelic or biallelic *DUOX2* variants exhibited TD, suggesting a potential link between *DUOX2* and thyroid dysgenesis in specific cases. A possible explanation is that the *DUOX* family exerts additional effects on thyroid development through interactions with other unidentified proteins. *DUOXA2* resides on chromosome 15 in a head-to-head orientation with *DUOX2* and plays a crucial role in the posttranslational processing and enzymatic activity of *DUOX2* (42, 43). Both *DUOXA2* and *DUOX2* are vital components of the NADPH oxidase family (44). The main role of *DUOXA2* is in assisting and facilitating the maturation and activation of *DUOX2* (12, 44). Variations in *DUOXA2* can impact the ability of the thyroid to generate H_2_O_2_, leading to disturbances in thyroid hormone synthesis (14). In our research, the *DUOXA2* gene variant detection rate was 8.3% (9/109), suggesting that in the local population, *DUOX2*, in conjunction with *DUOXA2*, plays a major role in the occurrence of CH.

Several studies have proposed that CH patients with monoallelic *DUOX2* variants tend to exhibit TCH and that those with biallelic *DUOX2* variants are more likely to experience PCH (45). Our findings did not reveal significant differences in biochemical results, initial L-T4 doses, or clinical outcomes between patients with monoallelic and biallelic *DUOX2* mutations. However, a significant association was found between *DUOX2* biallelic variants and goiter, with monoallelic *DUOX2* variants often associated with normal thyroid morphology. The above findings suggest a strong correlation between the quantity of *DUOX2* variations and thyroid morphology. How to predict clinical outcomes based on these findings remains highly uncertain.

In general, the severity of the CH phenotype in relation to the quantity of CH gene variants remains unclear. Notably, this study revealed digenic variants in 11 patients and oligogenic variants in 3 patients. Among these gene combinations, *DUOX2*+*TG* was most common, constituting 27.3% (3/11). Wang et al. identified the most common digenic variant as *DUOX2*/*DUOXA1* in 61 CH patients, in contrast to our findings (46). This discrepancy might be attributed to the smaller sample size in our study and variations in geographical and ethnic factors. Prior research suggests that the more gene variant sites or types there are, the more challenging it is for thyroid function to return to normal, requiring longer durations and larger doses of medication for maintenance (47, 48). In this study, we categorized CH patients into four groups based on the gene variant types and quantities. No statistically significant differences were observed among these groups regarding biochemical results, initial L-T4 doses or clinical outcomes. However, concerning thyroid morphology, the “monoallelic variant group” predominantly exhibited normal morphology, whereas goiters became more prevalent with an increase in gene variant types and quantities. These findings suggest that synergistic interactions between gene variants enhance the negative feedback loop of the hypothalamus-pituitary-thyroid axis, leading to elevated serum TSH levels, a gradual increase in thyroid volume, and eventual goiter. Despite these morphological differences, no significant differences were observed in clinical outcomes among the four groups, with PCH remaining the primary clinical outcome. A subset of 25 CH patients underwent three years of standardized follow-up, with no significant differences observed in physical or intellectual development assessments at 1, 2, and 3 years of age among patients with different gene types and quantities. Although the sample size is limited, these findings emphasize the paramount role of standardized L-T4 treatment and follow-up in influencing treatment outcomes for CH children.

Recent studies have increasingly highlighted the role of digenic and oligogenic inheritance patterns in CH, suggesting that combined variants in *DUOX2*, *DUOXA2*, *TG*, and other genes may exert synergistic effects on thyroid development and function. These findings reinforce our observation of multi-gene variants in a subset of patients and further underscore the complexity of genotype–phenotype correlations in CH (49). Integrating these recent insights, it becomes evident that comprehensive genetic screening and careful interpretation are essential, and that functional validation will be particularly important for variants of uncertain significance detected in oligogenic contexts (50).

Consistent with other East Asian cohorts, our Yunnan series showed a *DUOX2*-dominant genetic spectrum of primary CH. Population-based and hospital-based studies from mainland China similarly report dyshormonogenesis as the major etiology with *DUOX2* being the most frequently mutated gene, often accompanied by *DUOXA2/TG/TPO* variants and occasional oligogenic patterns (30). In Korea, *DUOX2* mutations are likewise a frequent cause with several recurrent alleles, supporting a shared East-Asian pattern (36). apanese data using targeted NGS panels also identify recurrent CH-associated variants detectable by screening panels, in line with an East-Asian mutation spectrum (37). In contrast, studies from consanguineous populations in the Middle East and from Sudan report a predominance of *TPO* and *TG* mutations among dyshormonogenesis cases, underscoring population-specific architectures likely shaped by founder effects and consanguinity (38). These comparisons suggest that our findings are generalizable within East Asia but may not extend to regions where *TPO/TG* defects prevail; this population contrast has been emphasized in recent reviews and consensus statements (14).

This study has several limitations. First, the relatively small sample size implies that the conclusions drawn may not comprehensively explain the genotype–phenotype correlation in CH patients. Future research should aim to expand the sample size for more robust analysis. Second, the CH patients in this study were exclusively from Yunnan Province. Given regional and ethnic differences, the findings of this study may not be representative of the entire country of mainland China, highlighting the need for multicenter prospective studies. Finally, further functional studies are warranted to validate the pathogenicity of these variants of uncertain significance (VUSs), enhancing the clarity of the genotype–phenotype relationship.

In conclusion, CH is a complex endocrine disorder, and although efforts have been made to decipher its genetic code, the relationship between genotype and phenotype is not straightforward. Possible genetic causes identified through NGS may not be possible for all CH patients, and unknown genes or environmental factors may play a role. Currently, supported by governmental initiatives, establishment of a standardized system for screening, recall, diagnosis, treatment and follow-up in newborn screening centers is crucial for ensuring the normal growth and development of CH children.

## Data Availability

The original contributions presented in the study are included in the article/**Supplementary Material**. Further inquiries can be directed to the corresponding authors.

## Supplementary material

Supplementary Table 1 Clinical and molecular characteristics of 91 children with congenital hypothyroidism. M, male; F, female; SHT, subclinical hypothyroidism; CHT, clinical hypothyroidism; Y, years; M, months; NBS, newborn screening; TCH, transient congenital hypothyroidism; PCH, permanent congenital hypothyroidism; P, pathogenic; LP, likely pathogenic; VUS, variant of uncertain significance.
