# Supplementary material for: Revealing the genotype-phenotype correlations of congenital hypothyroidism in Yunnan Province, Southwest China
Source: Front Endocrinol (Lausanne). 2025 Nov 17;16:1640108. doi: 10.3389/fendo.2025.1640108 (PMC12666528; doi:10.3389/fendo.2025.1640108)
Supplement: Supplementary Table 1 — Clinical and molecular characteristics of 91 children with congenital hypothyroidism. M, male; F, female; SHT, subclinical hypothyroidism; CHT, clinical hypothyroidism; Y, years; M, months; NBS, newborn screening; TCH, transient congenital hypothyroidism; PCH, permanent congenital hypothyroidism; P, pathogenic; LP, likely pathogenic; VUS, variant of uncertain significance. [file Table1.docx]

**Table S1. The clinical and molecular characteristics of 91 children with congenital hypothyroidism**

| **Case ID** | **Gender** | **Maternal thyroid function** | **Age**  **(Years)** | **Birth height**  **(cm)** | **Birth weight**  **(g)** | **Age of diagnosis（days）** | **TSH level at NBS (Reference value: <8 mIU/L）** | **Diagnostic serum TSH level (Reference value: 0.87–6.15 mIU/L)** | **Diagnostic serum FT4 level(Reference value: 12.0–18.6 pmol/L)** | **Thyroid morphology** | **Clinical outcome** | **Initial L-T4 dose (μg/day)** | **Maintenance L-T4 dose (μg/day)** | **Gene** | **Exon/IVS**  **position** | **Variants** | **Source** | **ACMG classification** |
| --- | --- | --- | --- | --- | --- | --- | --- | --- | --- | --- | --- | --- | --- | --- | --- | --- | --- | --- |
| 1 | M | Normal | 4Y7M | 50 | 3000 | 27 | 333 | 118 | 4.8 | Goiter | PCH | 37.5 | 50 | *DUOX2* | exon30 | c.3974A>G (p.H1325R) | Maternal | VUS |
|  |  |  |  |  |  |  |  |  |  |  |  |  |  | *PAX8* | exon3 | c.164A>G (p.H55R) | Paternal | VUS |
| 2 | F | Normal | 8Y2M | 51 | 3200 | 14 | 20 | 13 | 5.91 | Normal | PCH | 12.5 | 25 | *DUOX2* | exon13 | c.1462G>A (p.G488R) | Maternal | LP |
| 3 | M | CHT | 9Y3M | 50 | 3230 | 17 | 97.8 | 100 | 11.48 | Normal | PCH | 37.5 | 75 | *DUOX2* | exon6 | c.541G>A (p.G181S) | Maternal | VUS |
| 4 | F | Normal | 6Y3M | 50 | 2900 | 21 | 20 | 17 | 5 | Normal | PCH | 12.5 | 12.5 | *DUOX2* | exon20 | c.2654G>T (p.R885L) | Maternal | LP |
| 5 | F | Normal | 6Y6M | 50 | 2900 | 20 | 103 | 98 | 5.4 | Athyreosis | PCH | 37.5 | 50 | *DUOX2* | exon17 | c.2054T>C (p.V685A) | Maternal | VUS |
| 6 | M | Normal | 3Y6M | 50 | 3450 | 31 | 133 | 163.36 | 6.57 | Athyreosis | PCH | 37.5 | 37.5 | *DUOX2* | exon20 | c.2654G>T (p.R885L) | Maternal | LP |
| 7 | M | CHT | 7Y5M | 50 | 3700 | 33 | 8.55 | 13.9 | 10.48 | Normal | PCH | 12.5 | 25 | *DUOX2* | exon20 | c.2635G>A (p.E879K) | Maternal | P |
| 8 | M | Normal | 4Y5M | 51 | 3350 | 17 | 8.49 | 28.68 | 9.15 | Normal | PCH | 25 | 17 | *DUOX2* | exon20 | c.2654G>T (p.R885L) | Maternal | LP |
| 9 | M | Normal | 4Y6M | 50 | 3390 | 38 | 11.2 | 82.4 | 6.47 | Normal | PCH | 25 | 25 | *DUOX2* | exon17 | c.2048G>T (p.R683L) | Paternal | LP |
| 10 | F | Normal | 6Y10M | 50 | 3300 | 30 | 10.3 | 23 | 6.03 | Normal | TCH | 25 | 0 | *DUOX2* | exon20 | c.2654G>T (p.R885L) | Paternal | LP |
| 11 | M | Normal | 3Y11M | 50 | 2700 | 54 | 28 | 23.67 | 10.04 | Goiter | PCH | 25 | 25 | *DUOX2* | exon14 | c.1588A>T (p.K530X) | Paternal | P |
|  |  |  |  |  |  |  |  |  |  |  |  |  |  | *TG* | exon48 | c.8191G>A (p.G2731R) | Maternal | VUS |
| 12 | M | Normal | 4Y2M | 51 | 2850 | 37 | 8.53 | 26.96 | 7.5 | Goiter | TCH | 25 | 0 | *DUOX2* | exon10 | c.1097C>T (p.A366V) | Maternal | VUS |
| 13 | F | Normal | 6Y2M | 51 | 3000 | 18 | 47.4 | 100 | 1.08 | Goiter | PCH | 37.5 | 44 | *DUOX2* | exon20 | c.2654G>T (p.R885L) | Paternal | LP |
| 14 | M | Normal | 2Y8M | 48 | 2870 | 15 | 9.63 | 10.71 | 11.64 | Normal | TCH | 12.5 | 0 | *DUOX2* | exon14 | c.1633_1634insG (p.V545Gfs*7) | Paternal | P |
| 15 | M | CHT | 2Y5M | 51 | 3300 | 13 | 26.9 | 12.56 | 10.65 | Normal | TCH | 12.5 | 0 | *DUOX2* | IVS5 | c.514-12C>A | Paternal | VUS |
| 16 | F | Normal | 4Y8M | 50 | 2900 | 26 | 11.4 | 96.75 | 5.29 | Goiter | PCH | 25 | 25 | *GLIS3* | exon3 | c.430G>C (p.G144R) | Maternal | VUS |
| 17 | M | Normal | 2Y1M | 49 | 3200 | 21 | 12 | 14.5 | 11.15 | Normal | PCH | 12.5 | 20 | *DUOX2* | exon14 | c.1588A>T (p..K530X)) | Maternal | P |
| 18 | F | Normal | 3Y7M | 48 | 2200 | 29 | 9.67 | 83.18 | 3.48 | Goiter | PCH | 25 | 25 | *DUOX2* | exon26 | c.3478_3480del (p.L1160del) | Maternal | P |
| 19 | F | CHT | 4Y11M | 50 | 2900 | 20 | 69.3 | 100 | 4.2 | Goiter | PCH | 37.5 | 37.5 | *DUOX2* | exon26 | c.3478_3480del (p.L1160del) | Paternal | P |
|  |  |  |  |  |  |  |  |  |  |  |  |  |  | *TBL1X* | exon7 | c.480_481insGCGGCG (p.A168_T169insAA) | Maternal | P |
|  |  |  |  |  |  |  |  |  |  |  |  |  |  | *IGSF1* | exon9 | c.1630C>T (p.R544W) | Paternal | VUS |
| 20 | F | Normal | 3Y6M | 50 | 3170 | 20 | 14.8 | 58.18 | 8.36 | Hypoplasia | PCH | 25 | 25 | *DUOX2* | exon21 | c.2779A>G (p.M927V) | Maternal | VUS |
| 21 | M | SHT | 4Y1M | 50.6 | 3070 | 21 | 70.9 | 100 | 2.96 | Goiter | PCH | 37.5 | 25 | *DUOX2* | exon18 | c.2180delG(p.G727Afs*23) | Maternal | P |
|  |  |  |  |  |  |  |  |  |  |  |  |  |  |  | exon25 | c.3329G>A (p.R1110Q) | Paternal | P |
| 22 | F | Normal | 5Y6M | 49 | 2800 | 31 | 92.8 | 100 | 3.05 | Athyreosis | PCH | 37.5 | 30 | *DUOX2* | exon15 | c.1708C>T (p.Q570X) | Maternal | LP |
|  |  |  |  |  |  |  |  |  |  |  |  |  |  |  | exon20 | c.2654G>T (p.R885L) | Paternal | P |
|  |  |  |  |  |  |  |  |  |  |  |  |  |  | *IYD* | exon4 | c.599G>C (p.G200A) | Maternal | VUS |
| 23 | F | Normal | 6Y11M | 49.2 | 2900 | 19 | 17.9 | 71.9 | 6.11 | Goiter | PCH | 25 | 25 | *DUOX2* | exon14 | c.1588A>T (p.K530X) | Maternal | LP |
|  |  |  |  |  |  |  |  |  |  |  |  |  |  |  | exon28 | c.3693+1G>T | — | P |
|  |  |  |  |  |  |  |  |  |  |  |  |  |  | *TPO* | exon10 | c.1682C>T (p.T561M) | — | LP |
| 24 | M | Normal | 4Y | 49 | 2980 | 32 | 100 | 46.97 | 11.96 | Goiter | PCH | 25 | 30 | *DUOX2* | exon14 | c.1588A>T (p.K530X) | Paternal | P |
|  |  |  |  |  |  |  |  |  |  |  |  |  |  |  | exon25 | c.3329G>A (p.R1110Q) | Maternal | P |
| 25 | M | Normal | 9Y2M | 50.6 | 3510 | 25 | 27 | 17.43 | 6.98 | Normal | PCH | 12.5 | 25 | *DUOX2* | exon20 | c.2654G>T (p.R885L) | Maternal | P |
|  |  |  |  |  |  |  |  |  |  |  |  |  |  |  | exon24 | c.3061C>T (p.R1021X) | Paternal | LP |
|  |  |  |  |  |  |  |  |  |  |  |  |  |  | *TG* | exon44 | c.7753C>T (p.R2585W) | Maternal | VUS |
|  |  |  |  |  |  |  |  |  |  |  |  |  |  | *POU1F1* | exon3 | c.224C>A (p.T75N) | Paternal | VUS |
| 26 | M | Normal | 4Y6M | 51 | 3000 | 52 | 131 | 124.68 | 7.12 | Goiter | PCH | 37.5 | 29 | *DUOX2* | exon14 | c.1588A>T (p.K530X) | Paternal | LP |
|  |  |  |  |  |  |  |  |  |  |  |  |  |  |  | exon28 | c.3693+1G>T | Maternal | P |
| 27 | F | Normal | 9Y10M | 49.6 | 3000 | 15 | 237 | 14.2 | 8.17 | Goiter | PCH | 12.5 | 62.5 | *DUOX2* | exon22 | c.2921G>A (p.R974H) | Maternal | VUS |
|  |  |  |  |  |  |  |  |  |  |  |  |  |  |  | exon25 | c.3391G>T (p.A1131S) | Paternal | LP |
| 28 | F | Normal | 7Y4M | 49.4 | 2900 | 20 | 21 | 10 | 4.9 | Normal | PCH | 12.5 | 30 | *DUOX2* | exon13 | c.1546C>T (p.R516C) | Paternal | P |
|  |  |  |  |  |  |  |  |  |  |  |  |  |  |  | exon20 | c.2635G>A (p.E879K) | Maternal | LP |
| 29 | F | Normal | 9Y7M | 48.8 | 2950 | 20 | 28 | 9.8 | 5.1 | Goiter | PCH | 12.5 | 30 | *DUOX2* | exon13 | c.1462G>A (p.G488R) | Paternal | LP |
|  |  |  |  |  |  |  |  |  |  |  |  |  |  |  | exon17 | c.2048G>T (p.R683L) | Maternal | LP |
| 30 | M | Normal | 6Y1M | 51.2 | 3300 | 9 | 110 | 100 | 5.64 | Goiter | TCH | 37.5 | 0 | *DUOX2* | exon17 | c.2048G>T (p.R683L) | Maternal | LP |
|  |  |  |  |  |  |  |  |  |  |  |  |  |  |  | exon28 | c.3693+1G>T | Paternal | LP |
| 31 | F | Normal | 3Y10M | 51.2 | 3000 | 20 | 32.4 | 111.94 | 5.09 | Normal | PCH | 37.5 | 37.5 | *DUOX2* | exon14 | c.1588A>T (p.K530X) | Maternal | VUS |
|  |  |  |  |  |  |  |  |  |  |  |  |  |  |  | exon32 | c.4348T>C (p.Y1450H) | Paternal | P |
| 32 | M | Normal | 4Y1M | 50.3 | 3000 | 29 | 25.4 | 38.52 | 8.05 | Goiter | PCH | 25 | 50 | *DUOX2* | exon17 | c.2048G>T (p.R683L) | Paternal | VUS |
|  |  |  |  |  |  |  |  |  |  |  |  |  |  |  | exon34 | c.4537G>C (p.G1513R) | Maternal | LP |
| 33 | M | Normal | 5Y2M | 51.1 | 2800 | 14 | 60 | 55.1 | 4.41 | Normal | TCH | 25 | 0 | *DUOX2* | exon17 | c.2101C>T (p.R701X) | Paternal | LP |
|  |  |  |  |  |  |  |  |  |  |  |  |  |  |  | exon22 | c.2921G>A (p.R974H) | Maternal | P |
|  |  |  |  |  |  |  |  |  |  |  |  |  |  | *TRHR* | exon1 | c.493G>A (p.D165N) | Maternal | VUS |
| 34 | M | Normal | 6Y2M | 50.9 | 3350 | 20 | 15 | 13.08 | 11.03 | Normal | PCH | 12.5 | 37.5 | *DUOX2* | exon20 | c.2635G>A (p.E879K) | Maternal | LP |
|  |  |  |  |  |  |  |  |  |  |  |  |  |  |  | exon28 | c.3693+1G>T | Paternal | P |
| 35 | M | Normal | 5Y4M | 49.4 | 3200 | 19 | 51 | 11.32 | 10.57 | Normal | TCH | 12.5 | 0 | *DUOX2* | exon14 | c.1588A>T (p.K530X) | Paternal | P |
|  |  |  |  |  |  |  |  |  |  |  |  |  |  |  | exon25 | c.3329G>A (p.R1110Q) | Maternal | P |
| 36 | M | Normal | 4Y4M | 49.7 | 2950 | 28 | 26.7 | 16.12 | 9.7 | Goiter | TCH | 12.5 | 0 | *DUOX2* | exon14 | c.1588A>T (p.K530X) | Paternal | LP |
|  |  |  |  |  |  |  |  |  |  |  |  |  |  |  | exon20 | c.2654G>T (p.R885L) | Maternal | P |
|  |  |  |  |  |  |  |  |  |  |  |  |  |  | *LEPR* | exon20 | c.2948T>A (p.F983Y) | Maternal | VUS |
| 37 | F | Normal | 4Y4M | 49.6 | 2700 | 30 | 116 | 30.49 | 7.37 | Goiter | PCH | 25 | 30 | *DUOX2* | exon25 | c.3329G>A (p.R1110Q) | Maternal | LP |
|  |  |  |  |  |  |  |  |  |  |  |  |  |  |  | exon28 | c.3693+1G>T | Paternal | P |
| 38 | F | Normal | 3Y11M | 49.5 | 3200 | 19 | 10.5 | 23.35 | 9.48 | Goiter | TCH | 25 | 0 | *DUOX2* | exon10 | c.1126C>T (p.R376W) | Paternal | P |
|  |  |  |  |  |  |  |  |  |  |  |  |  |  |  | exon25 | c.3329G>A (p.R1110Q) | Maternal | P |
| 39 | M | Normal | 9Y3M | 50 | 3200 | 41 | 100 | 78.36 | 9.08 | Normal | PCH | 37.5 | 37.5 | *DUOX2* | exon12 | c.1295G>A (p.R432H) | Maternal | LP |
|  |  |  |  |  |  |  |  |  |  |  |  |  |  |  | exon20 | c.2654G>T (p.R885L) | Paternal | LP |
| 40 | F | SHT | 5Y6M | 48.8 | 2900 | 22 | 14.3 | 94.3 | 4.22 | Goiter | TCH | 37.5 | 0 | *DUOX2* | exon14 | c.1588A>T(p.K530X) | Maternal | P |
|  |  |  |  |  |  |  |  |  |  |  |  |  |  |  | exon25 | c.3329G>A (p.R1110Q) | Paternal | P |
| 41 | F | Normal | 6Y2M | 52 | 3600 | 16 | 22.2 | 14.96 | 11.08 | Normal | PCH | 12.5 | 17 | *DUOX2* | exon13 | c.1546C>T (p.R516C) | Paternal | P |
|  |  |  |  |  |  |  |  |  |  |  |  |  |  |  | exon14 | c.1588A>T (p.K530X) | Maternal | LP |
|  |  |  |  |  |  |  |  |  |  |  |  |  |  | *TG* | exon20 | c.4165G>T (p.A1389S) | Maternal | VUS |
| 42 | M | CHT | 4Y4M | 49.4 | 2900 | 32 | 15.5 | 100 | 5.9 | Goiter | PCH | 37.5 | 25 | *DUOX2* | exon14 | c.1588A>T (p.K530X) | Maternal | VUS |
|  |  |  |  |  |  |  |  |  |  |  |  |  |  |  | exon16 | c.1873C>T (p.R625X) | Paternal | P |
|  |  |  |  |  |  |  |  |  |  |  |  |  |  |  | exon32 | c.4318G>A (p.D1440N) | Paternal | P |
| 43 | F | Normal | 2Y6M | 52 | 3700 | 23 | 22.1 | 11.10 | 9.59 | Normal | TCH | 12.5 | 0 | *DUOX2* | exon32 | c.4348T>C (p.Y1450H) | Maternal | VUS |
|  |  |  |  |  |  |  |  |  |  |  |  |  |  |  | exon32 | c.4375G>A (p.D1459N) | Paternal | VUS |
| 44 | F | Normal | 2Y4M | 50 | 3030 | 27 | 9.33 | 17.25 | 7.24 | Goiter | PCH | 12.5 | 12.5 | *DUOX2* | exon20 | c.2654G>T (p.R885L) | Maternal | VUS |
|  |  |  |  |  |  |  |  |  |  |  |  |  |  |  | IVS26 | c.3515+5G>A | Paternal | LP |
| 45 | F | Normal | 2Y1M | 48 | 2700 | 27 | 9.07 | 103.22 | 6.14 | Goiter | PCH | 37.5 | 12.5 | *DUOX2* | exon14 | c.1588A>T (p.K530X) | Paternal | P |
|  |  |  |  |  |  |  |  |  |  |  |  |  |  |  | exon25 | c.3329G>A (p.R1110Q) | Maternal | P |
| 46 | M | Normal | 3Y9M | 50 | 3700 | 15 | 23.4 | 118.41 | 2.63 | Normal | PCH | 37.5 | 12.5 | *DUOX2* | exon16 | c.1883delA (p.K628Rfs*11) | Paternal | LP |
|  |  |  |  |  |  |  |  |  |  |  |  |  |  |  | exon20 | c.2654G>T (p.R885L) | Maternal | P |
| 47 | M | Normal | 2Y5M | 52 | 3600 | 54 | 25.7 | 14.76 | 9.12 | Goiter | PCH | 12.5 | 20 | *DUOX2* | exon14 | c.1588A>T (p.K530X) | Paternal | P |
|  |  |  |  |  |  |  |  |  |  |  |  |  |  |  | exon6 | c.605_621delAGCTGGCGTCGGGGCCC (p.Q202Rfs*93) | Maternal | P |
| 48 | F | Normal | 2Y3M | 51 | 3450 | 20 | 42 | 147.20 | 4.93 | Goiter | PCH | 37.5 | 50 | *DUOX2* | exon17 | c.2048G>T (p.R683L) | Maternal | LP |
|  |  |  |  |  |  |  |  |  |  |  |  |  |  |  | exon25 | c.3329G>A (p.R1110Q) | Paternal | P |
|  |  |  |  |  |  |  |  |  |  |  |  |  |  |  | exon30 | c.4027C>T (p.L1343F) | Maternal | LP |
| 49 | F | Normal | 2Y6M | 49 | 3350 | 31 | 16.6 | 49.05 | 9.2 | Goiter | PCH | 25 | 50 | *DUOX2* | exon12 | c.1268C>T (p.T423I) | Paternal | VUS |
|  |  |  |  |  |  |  |  |  |  |  |  |  |  |  | exon2 | c.41C>A (p.A14D) | Maternal | VUS |
| 50 | M | Normal | 2Y2M | 50 | 3000 | 30 | 8.63 | 9.93 | 5.80 | Normal | PCH | 10 | 20 | *DUOX2* | exon12 | c.1295G>A (p.R432H) | Paternal | P |
|  |  |  |  |  |  |  |  |  |  |  |  |  |  |  | exon16 | c.1871delG (p.G624Afs*15) | Maternal | LP |
| 51 | F | Normal | 6Y4M | 50 | 2900 | 40 | 20 | 112.83 | 3.99 | Normal | PCH | 37.5 | 37.5 | *DUOX2* | exon22 | c.2921G>A (p.R974H) | Paternal | LP |
|  |  |  |  |  |  |  |  |  |  |  |  |  |  |  | exon31 | c.4184T>A (p.I1395N) | Maternal | LP |
| 52 | F | Normal | 4Y10M | 49 | 2760 | 20 | 8 | 27.87 | 9.96 | Normal | PCH | 25 | 50 | *DUOX2* | exon6 | c.605_621delAGCTGGCGTCGGGGCCC (p.Q202Rfs*93) | Maternal | VUS |
|  |  |  |  |  |  |  |  |  |  |  |  |  |  |  | exon21 | c.2779A>G (p.M927V) | Paternal | P |
| 53 | M | Normal | 3Y7M | 49.3 | 2700 | 18 | 33.7 | 49.69 | 0.22 | Goiter | PCH | 25 | 20 | *DUOX2* | exon7 | c.871G>A (p.A291T) | Maternal | LP |
|  |  |  |  |  |  |  |  |  |  |  |  |  |  |  | exon17 | c.2048G>T (p.R683L) | Paternal | LP |
| 54 | F | Normal | 9Y2M | 50 | 3000 | 18 | 232 | 100 | 1.4 | Goiter | PCH | 37.5 | 56.25 | *DUOX2* | exon22 | c.2921G>A (p.R974H) | Paternal | LP |
|  |  |  |  |  |  |  |  |  |  |  |  |  |  |  | exon22 | c.2921G>A (p.R974H) | Maternal | LP |
| 55 | F | CHT | 4Y2M | 48 | 2800 | 18 | 114 | 187.5 | 2.42 | Goiter | PCH | 37.5 | 12.5 | *DUOX2* | exon20 | c.2654G>T (p.R885L) | Maternal | LP |
|  |  |  |  |  |  |  |  |  |  |  |  |  |  |  | exon28 | C.3693+1G>T | Paternal | LP |
| 56 | M | Normal | 2Y8M | 49 | 2940 | 14 | 76.7 | 50.5 | 1.25 | Goiter | PCH | 25 | 30 | *DUOX2* | exon20 | c.2635G>A (p.E879K) | Maternal | P |
|  |  |  |  |  |  |  |  |  |  |  |  |  |  |  | exon25 | c.3329G>A (p.R1110Q) | Paternal | P |
| 57 | M | Normal | 2Y2M | 47 | 1990 | 41 | 35 | 31.77 | 8.96 | Normal | PCH | 25 | 25 | *DUOX2* | exon14 | c.1588A>T (p.K530X) | Paternal | VUS |
|  |  |  |  |  |  |  |  |  |  |  |  |  |  |  | exon21 | c.2779A>G (p.M927V) | Maternal | P |
| 58 | M | Normal | 6Y9M | 48 | 1895 | 21 | 19.4 | 11.77 | 5 | Normal | PCH | 10 | 37.5 | *DUOX2* | exon14 | c.1588A>T (p.K530X) | Paternal | VUS |
|  |  |  |  |  |  |  |  |  |  |  |  |  |  |  | exon28 | c.3632G>A (p.R1211H) | Maternal | P |
| 59 | M | Normal | 2Y11M | 54 | 4000 | 31 | 41.3 | 150 | 4.87 | Goiter | PCH | 37.5 | 50 | *DUOX2* | exon17 | c.2048G>T (p.R683L) | Paternal | LP |
|  |  |  |  |  |  |  |  |  |  |  |  |  |  |  | exon30 | c.4027C>T (p.L1343F) | Maternal | LP |
| 60 | M | Normal | 4Y1M | 50 | 3500 | 22 | 143 | 187.5 | 3.8 | Goiter | PCH | 37.5 | 25 | *DUOX2* | exon16 | c.1883delA (p.K628Rfs*11) | Maternal | P |
|  |  |  |  |  |  |  |  |  |  |  |  |  |  |  | exon28 | c.3693+1G>T | Paternal | LP |
| 61 | F | Normal | 3Y | 52 | 3550 | 23 | 247 | 188.32 | 3.25 | Goiter | PCH | 37.5 | 25 | *DUOX2* | exon10 | c.1127G>T (p.R376L) | Maternal | VUS |
|  |  |  |  |  |  |  |  |  |  |  |  |  |  |  | exon25 | c.3329G>A (p.R1110Q) | Paternal | P |
| 62 | M | Normal | 3Y6M | 49 | 3600 | 18 | 28.2 | 14.92 | 6.61 | Normal | TCH | 12.5 | 0 | *DUOX2* | exon12 | c.1334T>C (p.L445P) | Paternal | VUS |
|  |  |  |  |  |  |  |  |  |  |  |  |  |  |  | exon17 | c.2048G>T (p.R683L) | Paternal | LP |
|  |  |  |  |  |  |  |  |  |  |  |  |  |  |  | exon30 | c.4027C>T (p.L1343F) | Maternal | LP |
| 63 | F | Normal | 6Y8M | 49 | 3200 | 35 | 52.7 | 100 | 7.07 | Goiter | PCH | 37.5 | 30 | *DUOX2* | exon4 | c.227C>T (p.P76L) | Paternal | P |
|  |  |  |  |  |  |  |  |  |  |  |  |  |  |  | exon27 | c.3516_3531del (p.K1174Sfs*12) | Maternal | LP |
| 64 | M | Normal | 8Y9M | 49 | 2950 | 20 | 37.2 | 100 | 4.71 | Goiter | PCH | 37.5 | 37.5 | *DUOX2* | exon4 | c.244C>G (p.R82G) | Paternal | LP |
|  |  |  |  |  |  |  |  |  |  |  |  |  |  |  | exon20 | c.2654G>A (p.R885Q) | Maternal | LP |
| 65 | M | Normal | 8Y8M | 50 | 2700 | 17 | 22 | 18.65 | 6.87 | Normal | PCH | 12.5 | 25 | *DUOX2* | exon16 | C.1868G>A (P.R623Q) | Paternal | P |
|  |  |  |  |  |  |  |  |  |  |  |  |  |  |  | exon16 | c.1871delG (p.G624Afs*15) | Maternal | P |
|  |  |  |  |  |  |  |  |  |  |  |  |  |  |  | exon20 | c.2635G>A (p.E879K) | Maternal | VUS |
| 66 | M | Normal | 7Y6M | 50 | 3600 | 20 | 10.2 | 64.51 | 8.22 | Normal | PCH | 25 | 25 | *DUOX2* | exon22 | c.2921G>A (p.R974H) | Paternal | P |
|  |  |  |  |  |  |  |  |  |  |  |  |  |  |  | exon25 | c.3329G>A (p.R1110Q) | Maternal | LP |
|  |  |  |  |  |  |  |  |  |  |  |  |  |  | *TSHR* | exon10 | c.2252A>G (p.K751R) | Paternal | VUS |
|  |  |  |  |  |  |  |  |  |  |  |  |  |  | *SLC26A4* | exon8 | c.919-2A>G | Paternal | P |
|  |  |  |  |  |  |  |  |  |  |  |  |  |  | *LHX3* | exon6 | c.979G>A (p.A327T) | Maternal | VUS |
| 67 | F | Normal | 7Y5M | 52 | 3540 | 24 | 182 | 19.6 | 8.8 | Goiter | PCH | 12.5 | 25 | *DUOX2* | exon11 | c.1232G>A (p.R411K) | Maternal | P |
|  |  |  |  |  |  |  |  |  |  |  |  |  |  |  | exon25 | c.3329G>A (p.R1110Q) | Paternal | VUS |
| 68 | F | SHT | 3Y11M | 51 | 3350 | 23 | 125 | 100 | 5.62 | Goiter | PCH | 37.5 | 37.5 | *TG* | exon8 | c.922G>C (p.A308P) | Maternal | LP |
|  |  |  |  |  |  |  |  |  |  |  |  |  |  |  | exon41 | c.7111C>T (p.R2371X) | Paternal | LP |
| 69 | M | Normal | 3Y6M | 50 | 2900 | 37 | 25.1 | 69.45 | 3.05 | Goiter | PCH | 25 | 25 | *TG* | exon24 | c.4859C>T (p.T1620M) | Maternal | LP |
|  |  |  |  |  |  |  |  |  |  |  |  |  |  |  | exon31 | c.5791A>G (p.I1931V) | Paternal | VUS |
|  |  |  |  |  |  |  |  |  |  |  |  |  |  | *DUOXA2* | exon5 | c.738C>G (p.Y246X) | Maternal | P |
| 70 | F | Normal | 3Y11M | 50 | 2600 | 24 | 9.96 | 6.67 | 8.1 | Normal | PCH | 10 | 25 | *TG* | exon18 | c.3885C>G (p.H1295Q) | Maternal | VUS |
|  |  |  |  |  |  |  |  |  |  |  |  |  |  | *GLIS3* | exon10 | c.2213C>T (p.S738F) | Maternal | VUS |
| 71 | F | Normal | 8Y9M | 49 | 2900 | 14 | 155 | 125 | 3.61 | Normal | PCH | 37.5 | 50 | *TPO* | exon9 | c.1471C>T (p.R491C) | Paternal | VUS |
|  |  |  |  |  |  |  |  |  |  |  |  |  |  |  | exon11 | c.1949G>A (p.G650E) | Maternal | VUS |
| 72 | M | Normal | 6Y6M | 51 | 3400 | 20 | 100 | 100 | 5.93 | Athyreosis | PCH | 37.5 | 70 | *TPO* | exon12 | c.2029G>A (p.V677I) | Paternal | VUS |
| 73 | F | Normal | 5Y6M | 52 | 2900 | 21 | 18.6 | 78.68 | 5.6 | Normal | PCH | 25 | 25 | *TPO* | exon9 | c.1385C>T (p.A462V) | Paternal | VUS |
| 74 | M | Normal | 7Y6M | 50 | 3050 | 20 | 280 | 100 | 3.76 | Goiter | PCH | 37.5 | 50 | *TPO* | exon15 | c.2578G>A (p.G860R) | Maternal | P |
|  |  |  |  |  |  |  |  |  |  |  |  |  |  | *DUOXA2* | exon4 | c.413dupA (p.Y138*) | Maternal | P |
| 75 | F | Normal | 4Y | 51 | 3300 | 40 | 42.8 | 56.69 | 7.78 | Normal | PCH | 25 | 25 | *TSHR* | exon10 | c.1295A>G (p.N432S) | Paternal | LP |
|  |  |  |  |  |  |  |  |  |  |  |  |  |  |  | exon10 | c.1555C>T (p.R519C) | Maternal | LP |
| 76 | F | Normal | 6Y7M | 51 | 2750 | 32 | 50 | 42.96 | 6.7 | Normal | PCH | 25 | 30 | *TSHR* | exon10 | c.1574T>C (p.F525S) | Maternal | VUS |
| 77 | F | Normal | 12Y2M | 50 | 3070 | 30 | 10 | 19.31 | 6.08 | Normal | PCH | 25 | 30 | *TSHR* | exon10 | c.1574T>C (p.F525S) | Paternal | VUS |
| 78 | F | Normal | 2Y7M | 50 | 3050 | 30 | 90.5 | 49.66 | 8.37 | Normal | PCH | 25 | 50 | *TSHR* | exon10 | c.1197C>G (p.T399T ) | Paternal | VUS |
|  |  |  |  |  |  |  |  |  |  |  |  |  |  |  | exon10 | c.1574T>C (p.F525S) | Maternal | VUS |
| 79 | F | Normal | 8Y6M | 50 | 3300 | 25 | 10 | 8.63 | 11.54 | Normal | PCH | 10 | 25 | *TSHR* | exon10 | c.1349G>A (p.R450H) | Paternal | P |
| 80 | F | Normal | 4Y4M | 50 | 3200 | 30 | 106 | 118.36 | 4.12 | Goiter | PCH | 37.5 | 30 | *DUOXA2* | exon5 | c.738C>G (p.Y246X) | Paternal | P |
|  |  |  |  |  |  |  |  |  |  |  |  |  |  | *TBL1X* | exon8 | c.624C>T (p.H208H) | Maternal | VUS |
| 81 | M | Normal | 8Y6M | 51 | 3150 | 30 | 25 | 16.22 | 9.23 | Normal | PCH | 12.5 | 30 | *DUOXA2* | exon3 | c.310G>C (p.G104R) | Maternal | VUS |
| 82 | F | SHT | 5Y10M | 51 | 3400 | 36 | 76.1 | 100 | 4.14 | Goiter | PCH | 37.5 | 30 | *DUOXA2* | exon5 | c.738C>G (p.Y246X) | Paternal | P |
| 83 | M | Normal | 2Y2M | 49 | 3460 | 25 | 69 | 39.7 | 10.51 | Goiter | PCH | 25 | 25 | *DUOXA2* | exon5 | c.738C>G (p.Y246X) | Maternal | P |
| 84 | M | Normal | 2Y4M | 50 | 3140 | 17 | 12.7 | 10.68 | 12 | Normal | TCH | 10 | 0 | *DUOXA2* | exon5 | c.738C>G (p.Y246X) | Maternal | P |
| 85 | M | Normal | 3Y7M | 50 | 2100 | 17 | 30.8 | 186.91 | 6.19 | Normal | PCH | 37.5 | 25 | *DUOXA2* | exon5 | c.738C>G (p.Y246X) | Maternal | P |
| 86 | F | Normal | 4Y11M | 50 | 2460 | 33 | 146 | 100 | 2.5 | Goiter | PCH | 37.5 | 44 | *DUOXA2* | exon5 | c.573G>A (p.W191X) | Paternal | LP |
|  |  |  |  |  |  |  |  |  |  |  |  |  |  |  | exon5 | c.738C>G (p.Y246X) | Maternal | P |
| 87 | F | CHT | 8Y | 51 | 3000 | 19 | 100 | 100 | 5.8 | Hypoplasia | PCH | 37.5 | 62.5 | *PAX8* | exon3 | c.91C>T (p.R31C) | Maternal | VUS |
| 88 | M | CHT | 5Y11M | 46 | 2400 | 23 | 177 | 100 | 4.01 | Goiter | PCH | 37.5 | 30 | *SLC5A5* | exon14 | c.1653C>T (p.G551G) | Paternal | VUS |
| 89 | M | SHT | 5Y10M | 49 | 3300 | 28 | 100 | 100 | 3.66 | Goiter | PCH | 37.5 | 50 | *CDCA8* | exon6 | c.400C>T (p.R134C) | Paternal | VUS |
| 90 | M | CHT | 3Y8M | 49 | 2900 | 11 | 420 | 52.62 | 1.72 | Goiter | PCH | 25 | 52.5 | *DUOX2* | IVS7 | c.883-82C>G (p.H669R) | Maternal | VUS |
| 91 | F | Normal | 8Y11M | 51 | 3350 | 45 | 19.3 | 6.37 | 6.8 | Hypoplasia | PCH | 12.5 | 62.5 | *LHX3* | exon6 | c.979G>A (p.A327T) | Maternal | VUS |

Abbreviations: M, male; F, female; SHT, subclinical hypothyroidism; CHT, clinical hypothyroidism; Y, year; M, month; NBS, Newborn screening; TCH, transient congenital hypothyroidism; PCH, permanent congenital hypothyroidism; P, pathogenic; LP, likely pathogenic; VUS, variants of uncertain significance.
